# Aldolase A Promotes Colorectal Cancer Progression through Targeting COPS6 and Regulating MAPK Signaling Pathway

**DOI:** 10.1155/2023/1702125

**Published:** 2023-07-06

**Authors:** Ya Lu, Yuan Zhang, Xinyue Wang, Hui Zhang, Yue Zhu, Junying Zhang, Huanhuan Sha, Renrui Zou, Yujie Gan, Ying Sui, Juan Wang, Tongde Du, Jianzhong Wu, Jifeng Feng

**Affiliations:** ^1^Jiangsu Cancer Hospital, Jiangsu Institute of Cancer Research, The Affiliated Cancer Hospital of Nanjing Medical University, Nanjing, China; ^2^Department of Endoscopy, Cancer Hospital of the University of Chinese Academy of Sciences (Zhejiang Cancer Hospital), Institute of Cancer and Basic Medicine (IBMC), China; ^3^Nanjing Jinling Hospital, China

## Abstract

Colorectal cancer (CRC) is a serious threat to human health, and its underlying mechanisms remain to be further explored. Aldolase A (ALDOA) has received increasing attention for its reported association with multiple cancers, but the role and mechanisms of ALDOA in CRC are still unclear. In the current study, high expression levels and enzymatic activity of ALDOA were detected in CRC tissues and cell lines, indicating the clinical significance of ALDOA in human CRC. In addition, silencing ALDOA significantly impaired the proliferation and metastasis of CRC cells *in vitro* and *in vivo*. Mechanistically, immunoprecipitation assays and mass spectrometry analysis identified the binding protein COPS6 of ALDOA. Furthermore, the promoting effects of upregulated ALDOA on CRC cell proliferation and metastasis were inhibited by COPS6 depletion, demonstrating COPS6 was required for ALDOA in mediating CRC progress. Moreover, the epithelial-mesenchymal transition (EMT) program and MAPK signaling pathway were found to be activated by ALDOA overexpression as well. In summary, our findings suggested that ALDOA facilitated the proliferation and metastasis of CRC by binding and regulating COPS6, inducing EMT, and activating the mitogen-activated protein kinase (MAPK) signaling pathway. The present study provided evidence for ALDOA as a promising potential biomarker for CRC.

## 1. Introduction

Colorectal cancer (CRC) is one of the most common cancers diagnosed in humans and the second leading cause of cancer death in the world [1]. It has risen to the third place for malignant tumors in China [2]. Patients with advanced CRC usually have poor prognosis due to the lack of early diagnostic biomarkers and effective treatments [3, 4]. Therefore, it is imperative to further explore the molecular mechanisms of CRC development and seek more reasonable and effective biological targets.

Increasing evidence has suggested that “cancer metabolism” is a new hallmark of cancer [5, 6]. Tumor cells tend to accelerate cancer progression through a more efficient glycolytic pathway [7], and thus, the key enzymes involved in glycolysis are of increasing interest [8]. More intriguingly, their nonenzymatic activity-dependent biological roles in carcinogenesis have also been gradually explored, such as the contribution of hexokinase 2 to KRas-driven lung cancer and ErbB2-driven breast cancer [9], the promotion of lactate dehydrogenase A in head and neck cancer metastasis [10], and the activation of PI3K/AKT and YAP/TAZ pathways in cancer cells by phosphofructokinase 1 [11], all of which were expected to be potential targets for cancer therapy.

Aldolase is the fourth enzyme in the glycolysis process, and its family members include aldolase A (ALDOA), aldolase B (ALDOB), and aldolase C (ALDOC), which are encoded by three different genes [12, 13]. ALDOA is a major functional member of the aldolase family due to its high activity and low *K*_*m*_ value [12]. It is also an object of increased interest because of its high expression in various malignancies [14–16]. Research has revealed the importance of ALDOA in cancers, but the underlying mechanisms remain to be elucidated.

The aim of the present study was to evaluate the biological significance and function of ALDOA in CRC. It was discovered that ALDOA overexpression and high enzyme activity stimulate CRC cell growth and metastasis.

## 2. Material and Methods

### 2.1. Patient Specimen Collection and Ethics Statement

CRC and adjacent normal tissue samples were collected from surgical resection of CRC patients at the Affiliated Cancer Hospital of Nanjing Medical University (Nanjing, China). CRC patients with any tumor stage and pathological grade (I-IV) were included, and patients who received neoadjuvant therapy were excluded. The CRC diagnosis was confirmed by clinical criteria and pathological analysis. Informed consent was obtained from all subjects. The clinical characteristics in 24 CRC patients are presented in Table 1. All study protocols were approved by the Ethics Committee of the Affiliated Cancer Hospital of Nanjing Medical University, China.

### 2.2. Aldolase Activity Assay

ALDOA enzymatic activity in CRC specimens and cell lines was measured using the Aldolase Activity Colorimetric Assay Kit (cat. no. K665-100; BioVision, Tucson, AZ, USA) according to the manufacturer's protocol. A total of 10 mg of CRC tissue samples were rapidly homogenized in 100 *μ*L of ice-cold Aldolase Assay Buffer and kept on ice for 10 min. After centrifuging at 10,000*g* for 5 min, 5 *μ*L of sample supernatant and 50 *μ*L of reaction mix were added to a 96-well plate (Corning, NY, USA). The absorbance (450 nm) was then immediately measured in kinetic mode for 45 min at 37°C. One unit of aldolase was the amount of enzyme that generated 1 *μ*mol of nicotinamide adenine dinucleotide per minute at pH 7.2 and 37°C.

### 2.3. Cell Lines and Culture Conditions

A total of five human CRC cell lines (DLD1, SW480, SW620, HCT116, and LoVo) and human normal colon epithelial cell line HCoEpiC used in the present study were purchased from the American Type Culture Collection. All cell lines were cultured in Dulbecco's Modified Eagle Medium (DMEM; KeyGEN BioTECH, Jiangsu, China) containing 10% fetal bovine serum (FBS; Gibco; Thermo Fisher Scientific, Inc., Waltham, USA), 100 U/mL penicillin, and 100 *μ*g/mL streptomycin (Invitrogen; Thermo Fisher Scientific, Inc., Waltham, USA) and maintained in 37°C incubators with 5% CO_2_.

### 2.4. RNA Extraction and qRT-PCR Analysis

Total RNA samples were extracted from tissues or cultured cells using TRIzol reagent (Invitrogen; Thermo Fisher Scientific, Inc.). The cDNA samples were synthesized using the Reverse Transcription Kit (TaKaRa, Tokyo, Japan) according to the manufacturer's guidelines. Quantitative real-time PCR (qRT-PCR) was used to detect the related genes using PowerUp SYBR Green Mix (Invitrogen; Thermo Fisher Scientific, Inc.). The 20 *μ*L reaction system consisted of 10 *μ*L of SYBR Green Mix, 1 *μ*L each of forward and reverse primers, 2 *μ*L of cDNA, and 6 *μ*L of double distilled water. Gene mRNA expression in cell lines was normalized to that of GAPDH and assessed via the 2^−*ΔΔ*Ct^ method. Gene mRNA levels in CRC tissues were normalized to the levels of 18S rRNA and calculated by the 2^−*Δ*Ct^ method. All qRT-PCR assays were performed in triplicate on ABI 7500 Fast Instrument (model 7300; Applied Biosystems; Thermo Fisher Scientific, Inc.). The primers were synthesized by Sangon Biotech (Shanghai, China). The primer sequences were as follows: ALDOA-F 5′ GGTGCTGGCTGCTGTCTACAAG 3′, ALDOA-R 5′ GACGCCTCCTCCTCACTCTGG 3′; ALDOB-F 5′ AAGGCCCTGAATGACCATCA 3′, ALDOB-R 5′ GCATTGAGGTTGAGAGTGGC 3′; ALDOC-F 5′ AACCTCAATGCCATCAACCG 3′, ALDOC-R 5′ GCTCCACCATCTTCTCCACT 3′; COPS6-F 5′ ACCCTATGACCAAGCACACA 3′, COPS6-R 5′ TGCTATCAGGTGTTCAGCCA 3′; GAPDH-F 5′ GGATTTGGTCGTATTGGGCG 3′, GAPDH-R 5′ ATCGCCCCACTTGATTTTGG 3′; and 18S-F 5′ CGAACGTCTGCCCTATCAACTT 3′, 18S-R 5′ ACCCGTGGTCACCATGGTA 3′.

### 2.5. Western Blotting Analysis and Antibodies

Protein samples were extracted from cells or tissues in RIPA lysis buffer (Invitrogen; Thermo Fisher Scientific, Inc.) containing a protease and phosphatase inhibitor cocktail (New Cell & Molecular Biotech Co., Ltd., Suzhou, China). Nucleus and cytoplasm proteins were isolated by the Nuclear and Cytoplasmic Protein Extraction Kit (cat. no. KGP150, KeyGEN BioTECH, Nanjing, China) according to the manufacturer's instruction. The amount of protein was quantified using the BCA Protein Assay Kit (Beyotime Biotechnology, Shanghai, China) according to the manufacturer's instructions. The protein extracts (10 *μ*g/sample) were then separated using 4–12% gradient sodium dodecyl sulfate polyacrylamide gel electrophoresis (SDS-PAGE, GenScript, Nanjing, China), transferred onto polyvinylidene difluoride membranes (Millipore, Bedford, MA), which were blocked with QuickBlock Blocking Buffer (Beyotime Biotechnology), and then incubated with primary and secondary antibodies (anti-mouse/rabbit IgG, HRP-linked antibody; cat. no. 7076/7074; 1 : 5000 dilution; Cell Signaling Technology, Boston, USA). Protein bands were visualized using an ECL chemiluminescence reagent (New Cell & Molecular Biotech Co., Ltd.) and an Odyssey imaging system (LI-COR Biosciences). Protein levels were normalized using tubulin (rabbit polyclonal; cat. no. 11224-1-AP; 1 : 2000 dilution; Proteintech Group, Wuhan, China). The band intensities were normalized using ImageJ software. The following antibodies were used: ALDOA (mouse monoclonal; cat. no. sc-390733; 1 : 1000 dilution; Santa Cruz, CA, USA), HRP-conjugated DYKDDDDK Tag (monoclonal; cat. no. HRP-66008; 1 : 5000 dilution; Proteintech Group), ALDOB (rabbit polyclonal; cat. no. 18065-1-AP; 1 : 1000 dilution; Proteintech Group), ALDOC (rabbit polyclonal; cat. no. 14884-1-AP; 1 : 1000 dilution; Proteintech Group), E-cadherin (rabbit monoclonal; cat. no. 3195; 1 : 1000 dilution; Cell Signaling Technology), N-cadherin (rabbit monoclonal; cat. no. 13116; 1 : 1000 dilution; Cell Signaling Technology), vimentin (rabbit monoclonal; cat. no. 5741; 1 : 1000 dilution; Cell Signaling Technology), p38 (rabbit monoclonal; cat. no. 8690; 1 : 1000 dilution; Cell Signaling Technology), p-p38 (rabbit monoclonal; cat. no. 8632; 1 : 1000 dilution; Cell Signaling Technology), ERK1/2 (rabbit monoclonal; cat. no. 4695; 1 : 1000 dilution; Cell Signaling Technology), p-ERK1/2 (rabbit monoclonal; cat. no. 4376; 1 : 1000 dilution; Cell Signaling Technology), ACTB (rabbit monoclonal; cat. no. AC038; 1 : 10000 dilution; ABclonal Technology, Wuhan, China), GAPDH (rabbit monoclonal; cat. no. 60004-1-Ig; 1 : 10000 dilution; Proteintech Group), lamin B1 (rabbit polyclonal; cat. no. 12987-1-AP; 1 : 2000 dilution; Proteintech Group), PKM (rabbit polyclonal; cat. no. 10078-2-AP; 1 : 1000 dilution; Proteintech Group), HSP90AB (rabbit polyclonal; cat. no. RK05737; 1 : 1000 dilution; ABclonal Technology), CSN6 (mouse monoclonal; cat. no. sc-393023; 1 : 1000 dilution; Santa Cruz, CA, USA), caspase-3 (rabbit monoclonal; cat. no. 9662; 1 : 1000 dilution; Cell Signaling Technology), and cleaved caspase-3 (rabbit monoclonal; cat. no. 9661; 1 : 1000 dilution; Cell Signaling Technology).

### 2.6. Immunofluorescence

For immunofluorescence (IF) testing, the cells were fixed with 4% paraformaldehyde (Sangon Biotech, Shanghai, China) for 10 min and then permeabilized with Triton X-100 (Invitrogen; Thermo Fisher Scientific, Inc.) for 15 min. ALDOA antibody (rabbit polyclonal; cat. no. A1142; 1 : 100 dilution; ABclonal Technology) was used for IF incubation, while 4,6-diamidino-2-phenylindole (Beyotime Biotechnology) was added for cell nucleus staining. The fluorescent images were then captured under a fluorescence microscope (Olympus Corporation, Tokyo, Japan).

### 2.7. Transfection of CRC Cells

Two individual ALDOA and one universal negative control small interfering RNA (siRNA) samples were purchased from RiboBio. The pcDNA3.1(+)-ALDOA overexpression plasmid was purchased from Public Protein/Plasmid Library (Jiangsu, China). The pcDNA3.1(+) empty vector was extracted using Endofree Plasmid Maxi Kit (Qiagen, Germany). The plasmid DNA or siRNAs were transiently transfected into CRC cells using Lipofectamine 3000 (Invitrogen; Thermo Fisher Scientific, Inc.) following the manufacturer's protocol. For details, cells were seeded into 6-well dishes to be 70-90% confluent at transfection and transfected with DNA/siRNA-lipid or siRNA-lipid complexes (without P3000 regent) that were incubating at room temperature for 10-15 min. The content of DNA/siRNAs was 2.5 *μ*g. The transfected cells were analyzed within 2-4 days of incubation at 37°C. The sequences of siRNAs were as follows: siALDOA-1: CCCAAGTTATCAAATCCAA and siALDOA-2: CCCTCTTCGTCTCTAACCA. siALDOA-2 was selected for follow-up experiments due to better knockdown efficiency.

### 2.8. Construction of Stable ALDOA Knockdown or Overexpression Cell Lines

Lentiviral particles underexpressing or overexpressing ALDOA, both tagged with green fluorescent protein, were purchased from Corues Biotechnology (Jiangsu, China). FLAG-tag was added to ALDOA-overexpressing lentiviral particles as well. These lentiviral particles were individually used to infect SW480 and DLD1 to generate the corresponding stable cell lines after puromycin (Thermo Fisher Scientific, Inc.) screening for one week. The scrambled shRNA or empty vector transfected cells were established as matched controls. The efficiency of ALDOA knockdown or overexpression was assessed using western blot and qRT-PCR assays.

### 2.9. Cell Proliferation Assay

The cells were seeded in 96-well plates at a density of 3000 cells per well. Cell viability was evaluated using Cell Counting Kit-8 (CCK-8; Dojindo Laboratories, Tokyo, Japan) daily for three days. Then, 10% CCK-8 solution was added to each well, and the cells were incubated for 1 h. The absorbance was measured at a wavelength of 450 nm on SpectraMax (Molecular Devices). Cell proliferation was tested by using the 5-ethynyl-2′-deoxyuridine (EdU) kit (RiboBio, Guangzhou, China) according to the manufacturer's protocol. The images were captured with a fluorescence microscope (Olympus Corporation, Tokyo, Japan).

### 2.10. Wound Healing Assay

The cells were cultured in six-well plates at a density of 5 × 10^5^ cells/mL. After the density reaching almost 100%, 1 *μ*g/mL mitomycin C (Sigma-Aldrich) was added for 2 h, and then, a 10 *μ*L sterile pipette tip was used to make a wound. The supernatant was then discarded after washing with phosphate buffer saline (KeyGEN BioTECH). Serum-free DMEM was added to the six-well plates, and the wound closure was measured every 24 h until the scratch was completely closed. The images were acquired using an inverted light microscope.

### 2.11. Migration and Invasion Transwell Assays

Cell invasion assays were performed using transwell chambers (Corning, NY, USA) precoated with diluted Matrigel (1 : 8 dilution; BD Biocoat, Corning), while cell migration assays were completed without Matrigel. A total of 40,000 cells in 200 *μ*L of serum-free DMEM were seeded in the upper chambers, and the lower chambers were filled with DMEM containing 20% FBS. After 48 h of incubation at 37°C, the cells migrated or invaded into the lower surface were fixed with 4% paraformaldehyde (Beyotime Biotechnology) and visualized using crystal violet staining (Beyotime Biotechnology). The images of cells were captured at 40x using a light microscope, and five random fields were counted in each chamber. Three independent experiments were performed.

### 2.12. Cell Apoptosis Analysis Using Flow Cytometry

Cell apoptosis assay was performed with the Annexin V-FITC/PI Apoptosis Detection Kit (KeyGEN BioTECH, Nanjing, China) according to the manufacturer's instruction. After being stained with Annexin V-FITC and PI, the cell apoptosis was analyzed on a flow cytometer (FACScan; BD Biosciences, Franklin Lake, NJ, USA).

### 2.13. Immunoprecipitation Assay and Mass Spectrometry Analysis

Immunoprecipitation (IP) assays were performed using the Dynabeads Protein G IP Kit (Thermo Fisher Scientific, Inc.) following the manufacturer's instructions. Anti-FLAG beads (Thermo Fisher Scientific, Inc.), stable ALDOA overexpression cell lines tagged with FLAG and their control cells, anti-ALDOA antibody (mouse monoclonal; cat. no. sc-390733; Santa Cruz, CA, USA), and anti-COPS6 antibody (mouse monoclonal; cat. no. sc-393023; Santa Cruz, CA, USA) were used in the IP assay. After the IP assay, 10% SDS-PAGE gel electrophoresis was performed and a silver stain was applied using the Fast Silver Stain Kit (Beyotime Biotechnology) following the manufacturer's instructions. Proteins contained in the specific silver staining bands were identified using mass spectrometry (MS).

### 2.14. Mouse Xenograft Model and Tail Vein Metastasis Model

All BALB/c nude mice (4–6 weeks old) were purchased from Charles River Laboratory (Beijing, China) for CRC *in vivo* experiments. In order to establish xenograft tumor models, the nude mice were randomly assigned into two groups with each group containing 6 female and 4 male mice. A total of 6 × 10^6^ cells (150 *μ*L) from each group (shCtrl or shALDOA) were subcutaneously injected into the side of each nude mouse. The tumor volumes and body weight of nude mice were observed and monitored every three days. The tumor volume was calculated using the following formula: volume (cm^3^) = (length × width^2^)/2. As tail vein cancer metastasis models, a total of 20 nude mice were randomized into two groups, five female and five male in each (shCtrl or shALDOA), and then injected via the tail vein with 5 × 10^5^ corresponding cells (100 *μ*L). After 30 days, the nude mice were placed into a container (clean and no CO_2_ prefilling) for euthanasia. Then, 100% CO_2_ was introduced into the container using compressed CO_2_ gas cylinder with a CO_2_ flow controller. The flow rate was 10-30% of the container volume per minute (1.25-3.75 L/min). The mice were then sacrificed via CO_2_ exposure for 2-3 min. After confirming that the nude mice were unconscious, on breathing, no heartbeat, and dilated pupils, the CO_2_ valve was closed, followed by observation for 2 min to ensure that the nude mice were dead. The subcutaneous tumors were immediately collected and measured, followed by hematoxylin-eosin (HE) staining and ki-67 detection (rabbit polyclonal; cat. no. 27309-1-AP; 1 : 8000 dilution; Proteintech Group). The lungs with metastasis foci were obtained and counted from tail vein metastasis models. All animal experiments were approved by the Institutional Animal Care and Use Committee of Nanjing Medical University, Nanjing, China (no. 2006034).

### 2.15. Statistical Analysis

Data that were independently collected in triplicate are presented as means ± standard deviation. Appropriate statistical methods including chi-square test, Fisher's exact test, one-way analysis of variance, and Student's *t*-test were used to calculate differences between groups with GraphPad Prism 7.0 software (GraphPad Software, Inc.). A *P* value of < 0.05 was considered statistically significant.

## 3. Results

### 3.1. ALDOA Is Upregulated in CRC Tissues and Cell Lines

**Figure 1:**
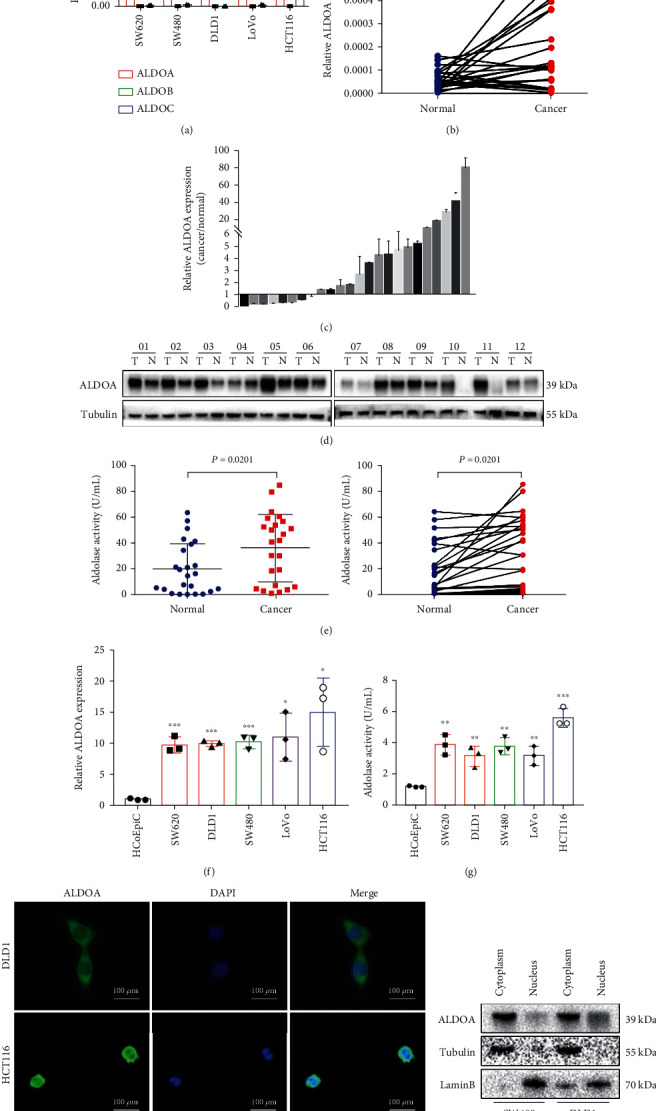
ALDOA was overexpressed in CRC tissues and cell lines. (a) ALDOA, ALDOB, and ALDOC were examined by qRT-PCR in CRC cell lines, and ALDOA occupied the most expression in aldolase family. (b, c) The overexpressed mRNA expression of ALDOA was tested in CRC tissues. (d) Western blotting showed elevated protein expression of ALDOA in CRC tissues. (e) The enzyme activity of ALDOA in CRC tissues was assessed higher level than normal. (f) Upregulation of ALDOA mRNA levels in CRC cell lines (SW620, DLD1, SW480, LoVo, and HCT116) was examined by qRT-PCR. (g) The ALDOA enzymatic activity was evaluated in CRC and normal colon epithelial cell lines. (h) The localization of ALDOA was observed mainly in cytoplasm by IF assay. (i) Western blotting detected ALDOA protein mainly expressed in cytoplasm. ^∗^*P* < 0.05, ^∗∗^*P* < 0.01, and ^∗∗∗^*P* < 0.001.

The qRT-PCR results showed that ALDOA was the main form of aldolase subtype in CRC cell lines (DLD1, SW480, SW620, HCT116, and LoVo) compared to ALDOB and ALDOC (Figure 1(a)). The mRNA and protein expressions of ALDOA, ALDOB, and ALDOC were then assessed in CRC tissues (*N* = 24). High ALDOA expression was detected both at mRNA and protein levels (Figures 1(b)–1(d)), but no significant differences in ALDOB or ALDOC were detected in CRC tissues (Figure S1A-C). In addition, there was positive correlation between ALDOA expression and depth of tumor invasion, lymph node metastasis, and TNM stage (Table 1). Moreover, ALDOA enzyme activity in CRC tissues was significantly higher than that in the corresponding normal tissues (Figure 1(e)). Compared to normal colon epithelial cells (HcoEpiC), the higher expression and enzyme activity of ALDOA were detected in CRC cell lines (Figures 1(f) and 1(g)). In addition, IF and western blotting results showed that ALDOA was mainly expressed in the cytoplasm (Figures 1(h) and 1(i)). These results revealed that ALDOA might be associated with CRC tumorigenesis.

### 3.2. ALDOA Knockdown Inhibits CRC Cell Proliferation, Invasion, and Migration *In Vitro*

**Figure 2:**
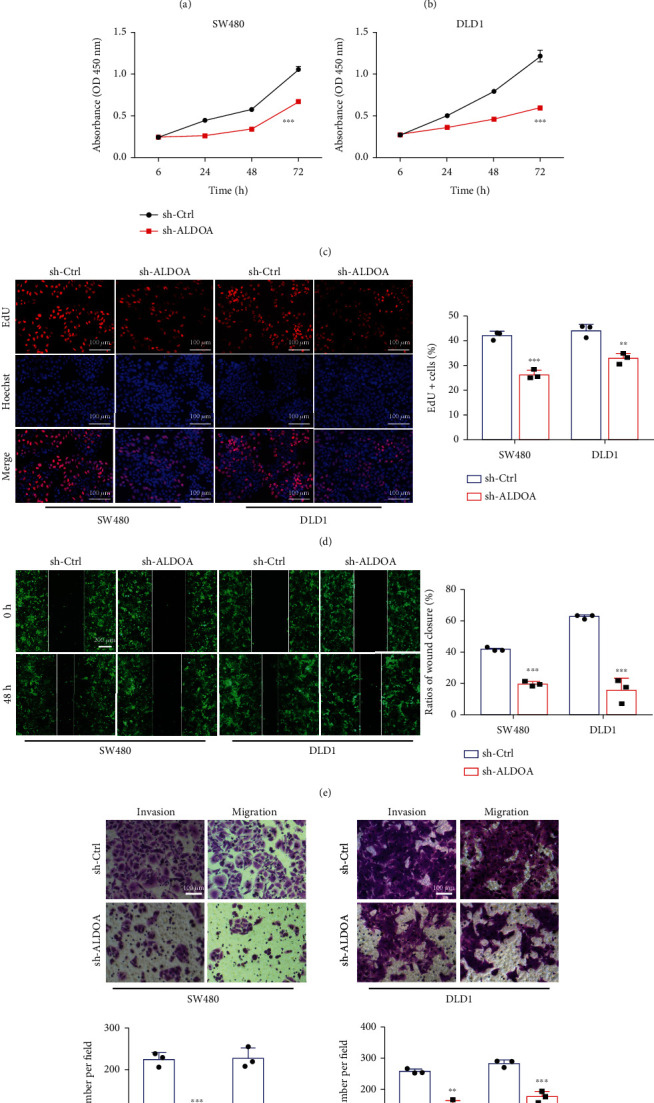
Knockdown ALDOA inhibited proliferation and metastasis of CRC cells *in vitro*. (a, b) The qRT-PCR and western blotting analyses were used to test the knockdown efficiency of ALDOA. (c) Knockdown of ALDOA suppressed proliferation activity of SW480 and DLD1 cells using CCK-8 assays. (d) EdU assays showed that CRC cell proliferation was inhibited by knockdown ALDOA. (e) ALDOA shRNA inhibited CRC cell migration according to wound healing assays. (f) Transwell assays, with Matrigel or not, were applied to assess the capability of CRC cells on invasion and migration. ^∗^*P* < 0.05, ^∗∗^*P* < 0.01, and ^∗∗∗^*P* < 0.001.

In previous studies, little was known about the potential biological functions of ALDOA in CRC. For subsequent experiments, stable ALDOA knockdown CRC cell lines were constructed in SW480 and DLD1. The efficiency of ALDOA was assessed both at mRNA and protein levels (Figures 2(a) and 2(b)). Next, results of the CCK-8 (Figure 2(c)), EdU (Figure 2(d)), wound healing (Figure 2(e)), and invasion and migration transwell (Figure 2(f)) assays showed that CRC cell proliferation, invasion, and migration abilities were significantly weakened after ALDOA was knocked down. However, flow cytometry and western blotting results showed that no significant difference was detected in apoptosis level in SW480 or DLD1 with stable knockdown ALDOA (Figure S2A, B). At the same time, the inhibitory effect of ALDOA on proliferation and metastasis of SW480 and DLD1 was also evaluated after transfection with ALDOA siRNA (Figure S3A-E). These data suggested that ALDOA served as an oncogene in the tumorigenesis and metastasis of CRC cells.

### 3.3. ALDOA Knockdown Reduces CRC Tumor Growth and Metastasis *In Vivo*

**Figure 3:**
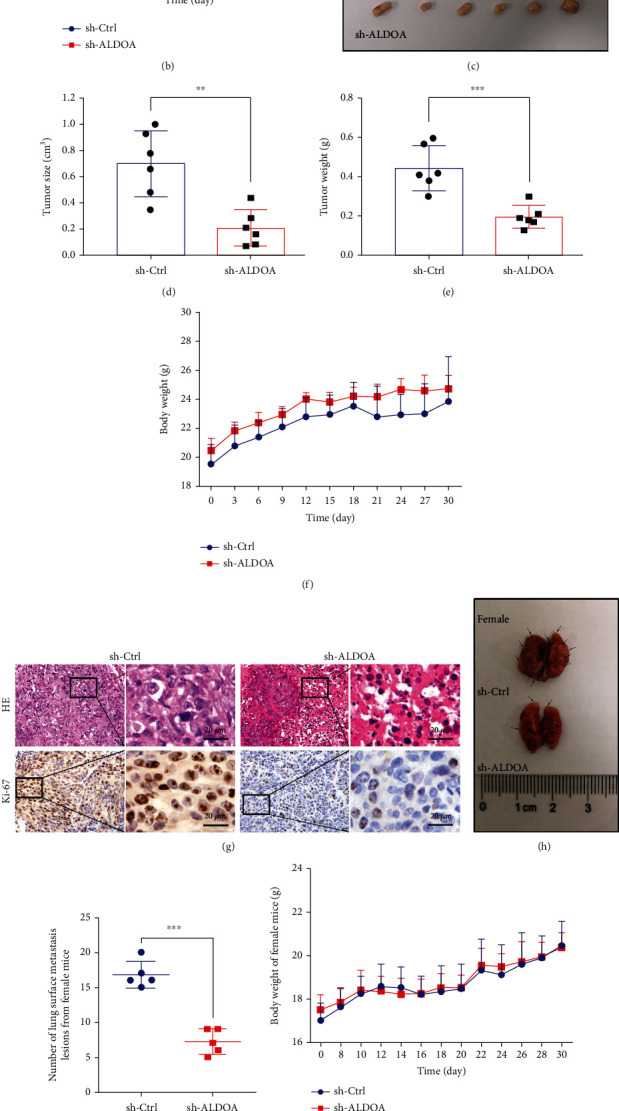
ALDOA facilitated CRC growth and metastasis *in vivo*. (a) Morphologic characteristics of CRC xenograft tumors on the surface of female nude mice. (b) The volume of subcutaneous tumors in mice was measured every three days. Volume = (length × width^2^)/2. (c) An intuitive morphology of tumors collected after mice was sacrificed. (d, e) Tumor volumes and weights at 30 day were measured in each group. (f) The nude mice were weighed every three days, but no significant difference was observed. (g) Representative images of HE and ki-67 staining in CRC xenograft tumors from sh-Ctrl and sh-ALDOA nude mice. (h, k) Knockdown ALDOA inhibited lung metastasis in nude mice. (i, l) The number of lung surface metastasis lesions was counted. (j, m) No significant difference was observed between CRC metastasis models. ^∗^*P* < 0.05, ^∗∗^*P* < 0.01, and ^∗∗∗^*P* < 0.001.

To determine whether ALDOA expression can affect CRC tumors *in vivo*, DLD1 cells with stable ALDOA knockdown or control cells were used to establish the xenograft models and the tail vein injection metastasis models. Consistent with the *in vitro* results, the stable ALDOA knockdown group showed remarkable inhibition of subcutaneous tumors compared to the control group (Figures 3(a)–3(e) and Figure S4A-D). Similarly, the number of lung metastasis in the mice with sh-ALDOA DLD1 cells was obviously decreased (Figures 3(h), 3(i), 3(k), and 3(l)). However, there was no statistically significant difference in body weight of nude mice between these paired groups (Figures 3(f), 3(j), and 3(m) and Figure S4E). In addition, HE and ki-67 staining showed lower growth activity of subcutaneous tumors from the ALDOA knockdown group (Figure 3(g)). Taken together, these results further indicated that ALDOA promoted CRC cell proliferation and metastasis *in vivo*.

### 3.4. ALDOA Overexpression Boosts CRC Cell Proliferation, Invasion, and Migration *In Vitro*

**Figure 4:**
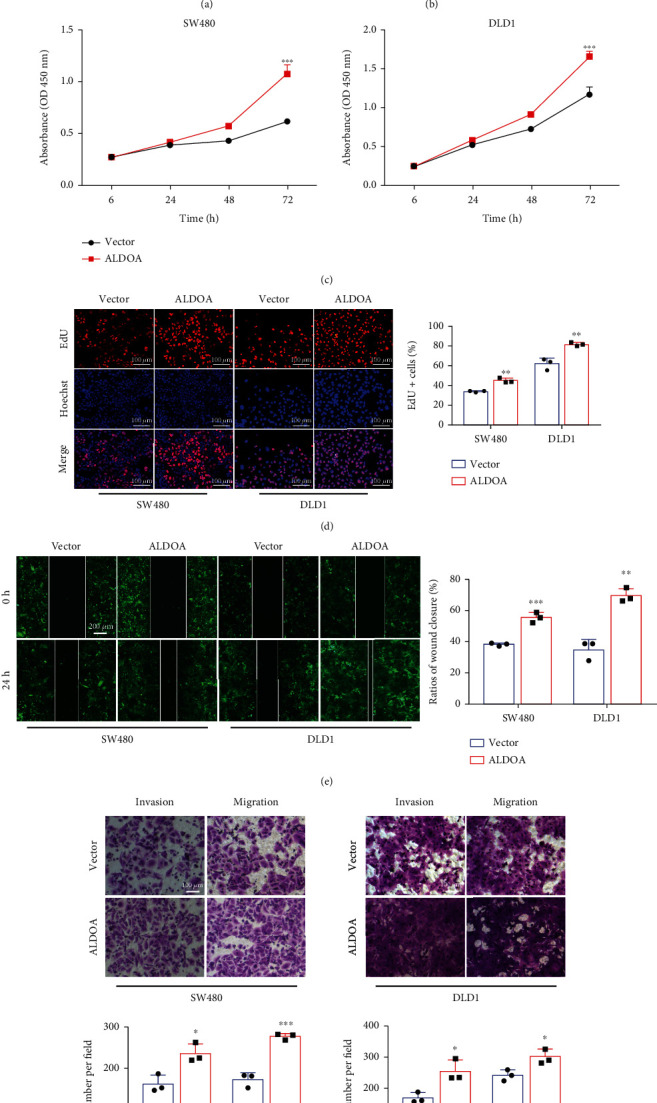
Overexpressed ALDOA enhanced the proliferation and metastasis of CRC cells. (a, b) The upregulated effect of stable ALDOA overexpression CRC cell lines was identified by qRT-PCR and western blotting analyses. (c) CCK-8 assays showed that upregulation of ALDOA promoted CRC cell proliferation. (d) Overexpressed ALDOA improved the ability of CRC cell proliferation using EdU assays. (e) Wound healing assays demonstrated that increased ALDOA was beneficial to CRC cell migration. (f) Matrigel-transwell and non-Matrigel-transwell assays, respectively, verified that overexpressed ALDOA enhanced the invasion and migration of CRC cells. ^∗^*P* < 0.05, ^∗∗^*P* < 0.01, and ^∗∗∗^*P* < 0.001.

In order to further explore the biological behavior of ALDOA in the progression of CRC, ALDOA-overexpressing CRC cell lines were constructed using the matched plasmid/lentivirus. Their efficiency was verified using qRT-PCR and western blotting assays (Figures 4(a) and 4(b) and Figure S5A, B). Eventually, consistent results were obtained in both transient and stable ALDOA overexpression cell lines. Upregulated ALDOA significantly enhanced the proliferation, invasion, and migration activity of CRC cells (Figures 4(c)–4(f) and Figure S5C-E) but had no significant effect on cell apoptosis level (Figure S2C, D). Therefore, the above results demonstrated that ALDOA performed an important function in facilitating CRC development.

### 3.5. ALDOA Binds to and Interacts with COPS6

**Figure 5:**
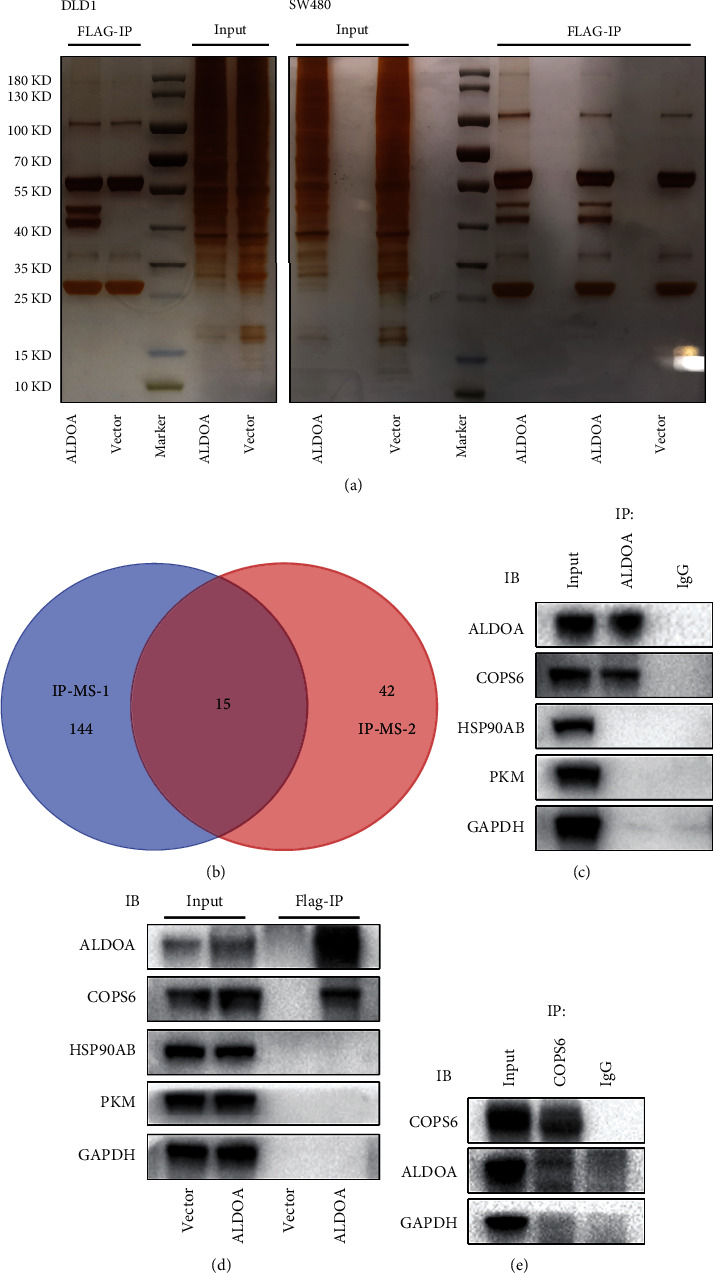
Protein-protein binding relationship between ALDOA and COPS6. (a) In stable overexpressed ALDOA cell lines tagged with flag, utilizing the characteristic of specific binding to flag beads, IP assays were performed to identify the interactive proteins that could bind to ALDOA. (b) Fifteen protein molecules and fragments were found to have the potential to bind to ALDOA according to the two MS analyses. (c–e) IP assays and western blotting were performed to confirm the binding relationship between ALDOA and COPS6.

Protein-protein interaction networks are important molecular mechanisms for protein-coding genes exerting biological roles. The IP assays, followed by MS analysis, were performed to identify the proteins that interact with ALDOA. In both SW480 and DLD1 cells, two protein bands interacted with flag-tagged ALDOA (Figure 5(a)). MS analysis identified a total of 15 proteins and peptide fragments as the potential binding proteins for ALDOA (Figure 5(b)). After performing the endogenous (anti-ALDOA/COPS6) and exogenous (anti-Flag) ALDOA Co-IP-western blotting assays, ubiquitin-related protein COPS6, a subunit of COP9 signalosome, was confirmed as a binding partner of ALDOA (Figures 5(c)–5(e)).

**Figure 6:**
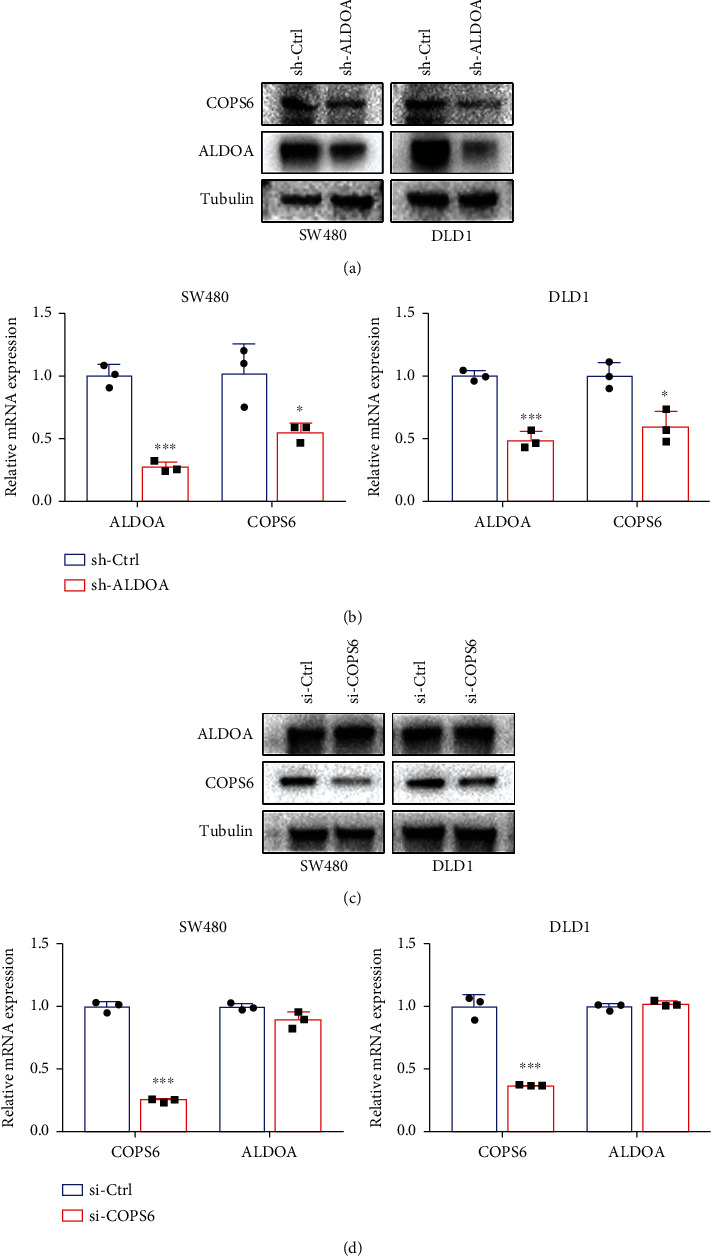
ALDOA regulated the expression of COPS6. (a, b) Western blotting and qRT-PCR analyses were used to assess the protein and mRNA levels of COPS6 in stable knockdown ALDOA cell lines. (c, d) The expression of ALDOA was detected by western blotting and qRT-PCR after COPS6 being knocked down with siRNA. ^∗^*P* < 0.05, ^∗∗^*P* < 0.01, and ^∗∗∗^*P* < 0.001.

Furthermore, qRT-PCR and western blotting results showed that mRNA and protein levels of COPS6 were reduced by ALDOA knockdown (Figures 6(a) and 6(b)), while the expression of ALDOA was not affected when COPS6 was knocked down (Figures 6(c) and 6(d)). These data suggested the possibility that ALDOA might influence the occurrence and development of CRC by interacting with COPS6.

In addition, further MS analysis of ALDOA modification revealed multiple ubiquitination modification sites (Table 2), indicating that the deubiquitination enzyme (DUB) COPS6 might stabilize the expression of ALDOA via the interactions between them.

### 3.6. COPS6 Is a Mediator of ALDOA's Roles in CRC

**Figure 7:**
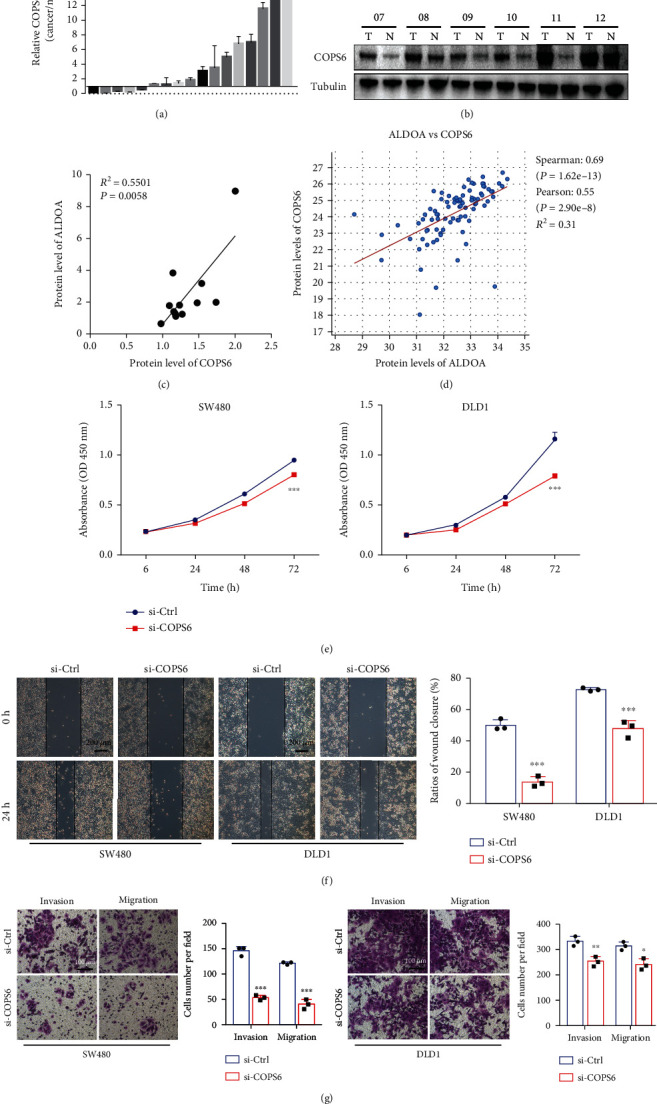
COPS6 acted as a cancer promoter in CRC. (a) The basal mRNA expression of COPS6 in CRC tissues was tested by qRT-PCR. (b) The protein expression of COPS6 in 12 paired CRC specimens was detected by western blotting. (c) Correlation analysis between COPS6 and ALDOA protein expression in CRC tissues. (d) Correlation analysis of ALDOA with COPS6 based on online databases (cBioPortal for Cancer Genomics, http://www.cbioportal.org/). (e) CCK-8 assays were used to evaluate the effect of COPS6 knockdown on the proliferation of SW480 and DLD1. (f) Wound healing assays were performed to assess the migration of CRC cell with COPS6 knockdown. (g) After COPS6 being knocked down by siRNA, cell transwell assays were applied to examine the invasion and migration of CRC cells. ^∗^*P* < 0.05, ^∗∗^*P* < 0.01, and ^∗∗∗^*P* < 0.001.

Based on the above findings, the biological function of COPS6 in CRC was clarified next. First, COPS6 was detected to be highly expressed in CRC tissues (Figures 7(a) and 7(b)). Furthermore, we found a positive correlation between COPS6 and ALDOA protein expressions in CRC tissues (Figure 7(c)), which was consistent with the database (http://www.cbioportal.org/) (Figure 7(d)). Then, the inhibition of proliferation, invasion, and migration of SW480 and DLD1 was detected after knocking down COPS6 with siRNA (Figures 7(e)–7(g)). A hypothesis was proposed, suggesting that ALDOA promotes CRC proliferation and metastasis by upregulating COPS6.

**Figure 8:**
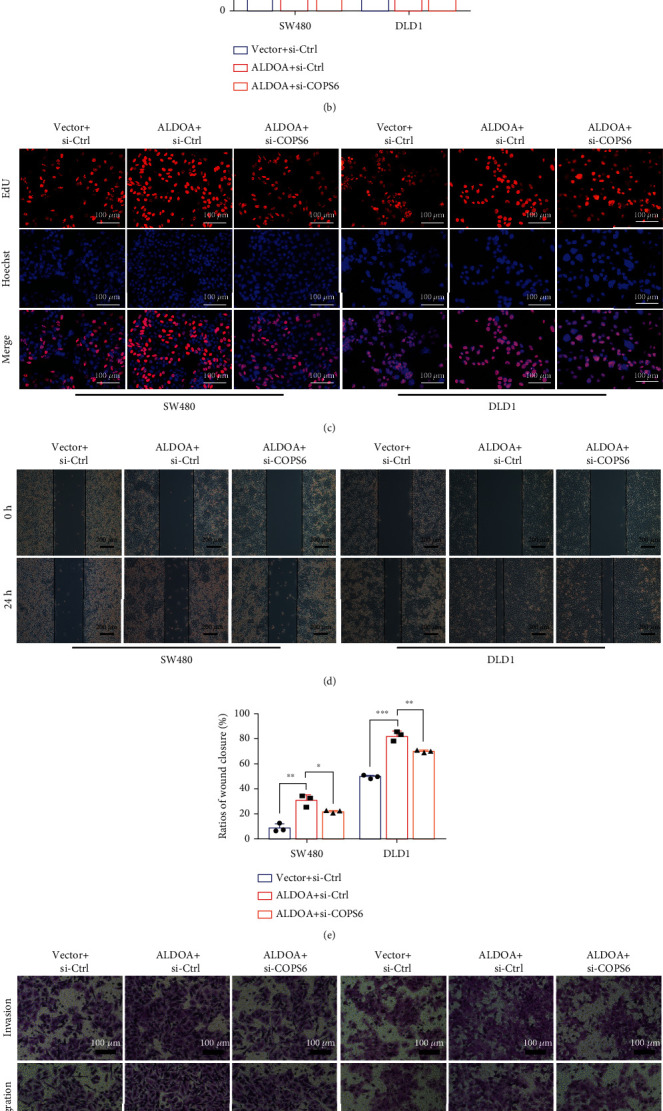
COPS6 knockdown reduced the promoting function of ALDOA in CRC cells. (a–c) CCK-8 and EdU assays showed that downregulated COPS6 weakens the promotion effect of ALDOA on CRC cell proliferation. (d–g) The migration and invasion of SW480 and DLD1 were tested and quantified by wound healing and transwell assays. The stable ALDOA overexpression CRC cell lines were transfected with siCOPS6 and siCtrl. ^∗^*P* < 0.05, ^∗∗^*P* < 0.01, and ^∗∗∗^*P* < 0.001.

Rescue assays were performed to explore whether COPS6 is the downstream mediator through which ALDOA affects CRC progression. Results showed that COPS6 knockdown suppressed the promotion of proliferation, invasion, and migration of CRC cells induced by overexpressed ALDOA (Figures 8(a)–8(g)). These data demonstrated that COPS6 served as a mediator of ALDOA to advance CRC progression.

### 3.7. ALDOA Promotes CRC Progression via EMT and MAPK Signaling Pathway

**Figure 9:**
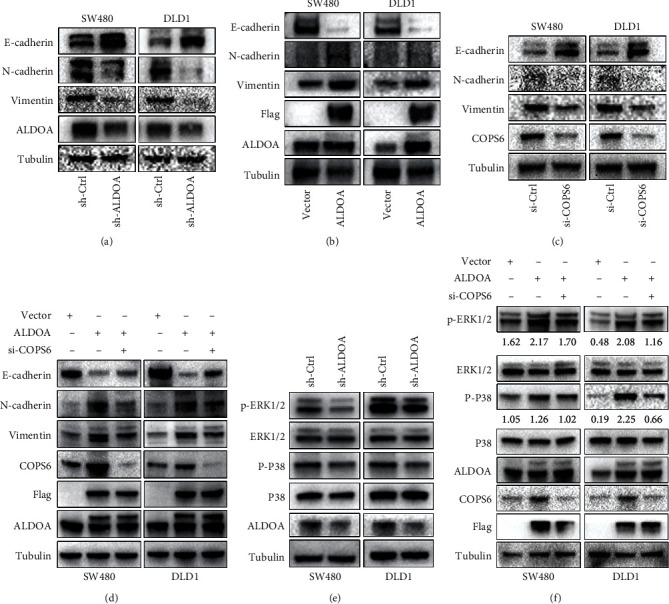
COPS6 was required for ALDOA accelerating CRC progression through EMT and MAPK signaling pathway. (a–d) Western blotting analysis was used to detect the expression of the indicated proteins in EMT program in CRC cells. (e, f) The expression of key proteins in MAPK signaling pathway was assessed by western blotting.

EMT is an important symbol of accelerating cancer progression and metastasis. It is also closely linked to the transformation of cytoskeletal proteins. The present study monitored the protein expression of EMT markers. Western blotting assays revealed that downregulated ALDOA or COPS6 markedly decreased the expression of N-cadherin and vimentin in both SW480 and DLD1, while the level of E-cadherin was increased (Figures 9(a) and 9(c)), and the opposite results were obtained when ALDOA was upregulated (Figure 9(b)). Furthermore, in order to clarify whether COPS6 is a key mediator of ALDOA promoting EMT in CRC cells, rescue assays were performed. The results showed that the downregulation of E-cadherin and the upregulation of N-cadherin and vimentin induced by ALDOA overexpression were all weakened by COPS6 knockdown (Figure 9(d)). These findings suggested that ALDOA could advance the EMT process of CRC cells, at least in part, through COPS6.

Furthermore, the relevant signaling pathways were examined to elucidate the specific molecular mechanisms of ALDOA in CRC. Results showed that ALDOA knockdown significantly reduced the level of phosphorylated p38 and extracellular signal-regulated kinase 1/2 (ERK1/2) in CRC cells (Figure 9(e)). The activation of p38 and ERK1/2 signals induced by overexpressed ALDOA was partially impaired by si-COPS6 (Figure 9(f)). This fully indicated that the promoting role of ALDOA in CRC progression by mediating COPS6 was associated with MAPK signaling pathway.

## 4. Discussion

The incidence and mortality of CRC have been increasing with changes in the aging population, dietary habits, gut microbiota status, and basic diseases [17, 18]. According to the latest global cancer statistics, CRC is the third most commonly diagnosed cancer (10.0% of the total cases) and the second leading cause of cancer deaths (9.4% of the total cancer deaths) in both sexes combined. The pathogenesis and molecular mechanism of CRC have not yet been elucidated. Further exploration should be carried out to find more appropriate biomarkers and therapeutic targets for clinical diagnosis and treatment of CRC.

Accumulating studies have reported that cell metabolism reprogramming is inextricably related to tumorigenesis [6, 19]. The increased lactic acid production and accelerated energy generation resulting from the glycolysis pathway create a favorable environment for cancer cell growth [7, 20]. ALDOA is a key enzyme in glycolysis that has been in the spotlight due to its association with the diagnosis, efficacy, and prognosis of multiple cancers [21–25]. However, the few previous studies on ALDOA in CRC have been mostly limited to correlation analysis, and little is known about its specific role and mechanism in CRC. The present study confirmed the higher expression and more enzyme activity of ALDOA in clinical specimens from CRC patients compared to corresponding normal tissues. Subsequently, biological function assays *in vitro* and *in vivo* were performed. ALDOA knockdown reduced CRC cell proliferation, invasion, and migration *in vitro*. Similarly, ALDOA silencing delayed the growth of subcutaneous CRC tumors in xenograft models and decreased the lung metastasis of CRC in tail vein injection metastasis models. Consistent with these results, CRC cell proliferation, invasion, and migration were enhanced with ALDOA upregulation. These outcomes provide additional evidence to support the role of ALDOA in facilitating CRC progression.

Furthermore, the potential mechanisms of ALDOA in CRC progression were explored as well. Although ALDOA is a glycolytic enzyme, it has been reported to function in a nonenzymatic manner in cancers. For example, Chang et al. have found that ALDOA expedited the acquisition of lung cancer stemness by suppressing miR-145 expression and activating the Oct4/DUSP4/TRAF4 axis in the absence of aldolase enzyme activity [26]. Additionally, aldolase isomers have been recognized as novel regulators of oncogenic Wnt signaling pathway due to their GSK-3*β* complex leading to Axin membrane translocation [27]. Notably, ALDOA established a cancer-associated protein-protein interaction with *γ*-actin, thereby influencing lung cancer cell metastasis [23]. Thus, IP assays and MS analysis were performed to better understand the molecular mechanisms behind ALDOA. The present study confirmed that ALDOA can interact with and regulate COPS6.

COPS6, also known as COP9 signalosome subunit 6 (CSN6), is a subunit of the COP9 signalosome [28]. Previous studies have shown that COPS6 was abnormally overexpressed in many cancers and usually predicted poor survival [29, 30]. This COPS6 property has also been reported in CRC [31, 32]. In CRC, CSN6 was deregulated by EGFR signaling, in which ERK2 bound directly to CSN6 Leu163/Val165 and phosphorylated CSN6 at Ser148. Then, CSN6 stabilized the *β*-catenin level by blocking the ubiquitin-proteasome pathway, thereby promoting CRC development [32]. The present study directly identified the role of COPS6 as a tumor promoter in CRC and as a downstream mediator of ALDOA in controlling CRC progression. At the same time, ALDOA expression was not influenced by COPS6. However, given that COPS6 is a DUB that has been widely reported to be involved in the ubiquitin-proteasome pathway, we proposed a hypothesis that COPS6 may stabilize ALDOA expression to some extent, thereby forming a positive feedback loop and further enhancing the impact on CRC progression. MS analysis of ALDOA modification sites revealed the presence of ubiquitination modification sites, which provided some evidence for this hypothesis. Moreover, in addition to ubiquitination modification sites, phosphorylation, acetylation, methylation, and other modification sites have also been found on ALDOA, indicating the existence of more diverse modification patterns. Further exploration and validation will help elucidate the molecular mechanisms of ALDOA in CRC.

EMT has long been proposed to be a crucial mechanism during cancer progression and metastasis, capable of bestowing cancer cells with the ability to spread throughout the body [33, 34]. During this process, cancer cells lose their epithelial characteristics and gain mesenchymal properties [35, 36], showing cell adhesion weakening and cell motility enhancement [37]. EMT is characterized by downregulation of epithelial cell junction proteins, for instance, E-cadherin, and activation of mesenchymal proteins, such as vimentin and N-cadherin [37]. The present study detected the increased expression of E-cadherin and decreased level of N-cadherin and vimentin in CRC cells with ALDOA and COPS6 knockdown. These results demonstrated that ALDOA and COPS6 facilitate CRC metastasis via EMT programs.

MAPKs are serine-threonine protein kinases that are involved in various cellular activities [38, 39]. The MAPK pathway is one of the most essential signaling cascades of tumorigenesis, including cancer cell proliferation, metastasis, and other biological behaviors [40, 41]. The MAPK signaling pathway includes p38 MAPK, c-Jun NH2-terminal kinase (JNK), and ERK in mammals [38]. Among them, the ERK MAPK pathway is the most important for cell proliferation and migration and is usually located downstream of many growth-related genes [40]. The p38 and JNK MAPK pathways are activated by multiple types of cellular stress and cointegration signals that affect proliferation, differentiation, survival, and migration [42]. Silenced ALDOA reduced the phosphorylation of p38 and ERK1/2, while activation of p38 and ERK1/2 caused by ALDOA overexpression was partially rescued by COPS6 knockdown. This illustrated that the MAPK signaling pathway is an important part of ALDOA that affects CRC development through COPS6.

**Figure 10:**
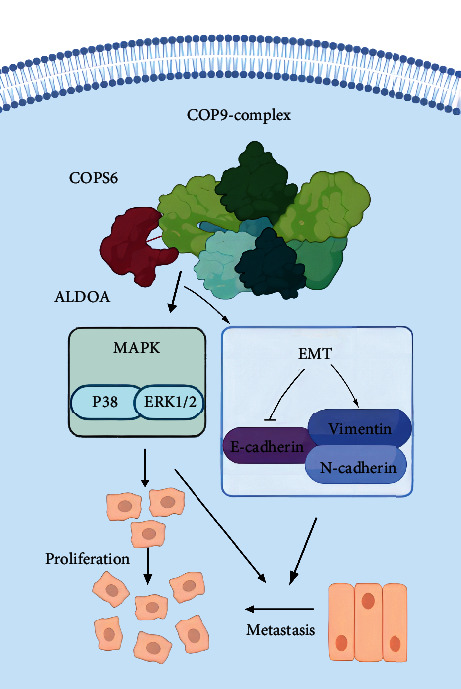
Graphical abstract. By binding to and targeting COPS6, aberrantly expressed ALDOA promoted the EMT process and activated the ERK1/2 and P38 signaling pathways, ultimately accelerating CRC cell proliferation and metastasis.

Taken together, these findings suggest that ALDOA overexpression promotes CRC cell proliferation and metastasis by interacting with COPS6, inducing EMT, and activating the MAPK signaling pathway (Figure 10). Future studies need to confirm the specific binding site between ALDOA and its target protein COPS6. Whether COPS6 influences the stability of ALDOA via a posttranslational mechanism also needs to be explored. In addition, a further investigation is required to describe the clinical application of ALDOA.

## 5. Conclusion

To summarize, aberrant ALDOA facilitated the EMT process, activated the MAPK signaling cascade of CRC cells by targeting COPS6, and ultimately accelerated CRC proliferation and metastasis. In conclusion, our study demonstrates the clinical and functional significance of ALDOA overexpression in CRC, potentially reserving a promising biomarker and therapeutic target for CRC.

## Figures and Tables

**Table 1:** Correlation between ALDOA expression and clinicopathological features of CRC patients.

Features	*N* of cases	ALDOA		*P* value
		High (*n*)	Low (*n*)	
Total	24	17	7	
Age (years)				
≤60	10	6	4	0.393
>60	14	11	3	
Gender				
Male	13	9	4	>0.999
Female	11	8	3	
Differentiation				
Well and moderately	11	7	4	0.659
Poorly	13	10	3	
Location				
Colon	13	9	4	>0.999
Rectum	11	8	3	
Depth of invasion				
T1	1	1	0	0.011^∗^
T2	3	0	3	
T3	12	8	4	
T4	8	8	0	
Lymph node metastasis				
Present	14	13	1	0.009^∗∗^
Absent	10	4	6	
Distant metastasis				
Present	4	4	0	0.283
Absent	20	13	7	
TNM stage				
I	4	1	3	0.033^∗^
II	6	3	3	
III	10	9	1	
IV	4	4	0	

^∗^ *P* < 0.05 and ^∗∗^*P* < 0.01.

**Table 2:** Posttranslational modification sites in ALDOA.

Modification	Sequence	MH+	Charge	Score	Expected value
GlyGly (K)	R.CQYVTEK#VLAAVYK.A	1785.92036	3	51.86	0.000000977
	R.ALQASALK#AWGGK.K	1414.78009	2	39.76	0.0000159
	K.VDK#GVVPLAGTNGETTTQGLDGLSER.C	2728.37481	3	39.08	0.0000309

Phospho (ST)	K.VDKGVVPLAGT^NGETTTQGLDGLSER.C	1691.84212	3	48.22	0.00000301
	K.GVVPLAGT^NGETTTQGLDGLSER.C	2352.10789	3	41.91	0.000178
	R.ALANS^LACQGK.Y	1212.54422	2	37.79	0.00000832

Acetyl (K)	K.DGADFAK^∗^WR.C	1107.52177	2	52.96	0.000000253

Methyl (DE)	R.IVAPGKGILAAD@E@STGSIAK.R	1926.0906	2	20.49	0.000893
	K.VD@KGVVPLAGTNGE@TTTQGLDGLSER.C	2642.3632	3	20.13	0.00243

## Data Availability

The data presented in this study are available on request from the corresponding author (J.F.: fjif@jszlyy.com.cn).

## Abbreviations

CRC: Colorectal cancer
ALDOA: Aldolase A
EMT: Epithelial-mesenchymal transition
MAPK: Mitogen-activated protein kinase
ALDOB: Aldolase B
ALDOC: Aldolase C
DMEM: Dulbecco's Modified Eagle Medium
FBS: Fetal bovine serum
qRT-PCR: Quantitative real-time PCR
SDS-PAGE: Sodium dodecyl sulfate polyacrylamide gel
IF: Immunofluorescence
siRNA: Small interfering RNA
CCK-8: Cell Counting Kit-8
EdU: 5-Ethynyl-2′-deoxyuridine
IP: Immunoprecipitation
MS: Mass spectrometry
HE: Hematoxylin-eosin
CSN6: COP9 signalosome subunit 6
ERK1/2: Extracellular signal-regulated kinase 1/2
JNK: c-Jun NH2-terminal kinase.

## Supplementary Materials

Supplementary Materials Figure S1: the expression of ALDOB and ALDOC in CRC tissues. (A) Western blotting and (B, C) qRT-PCR assays were used to assess the mRNA and protein levels of ALDOB and ALDOC in 24-paired CRC tissues. Figure S2: ALDOA had no significant effect on cell apoptosis. CRC cell apoptosis level was evaluated by (A, C) flow cytometry and (B, D) western blotting. Figure S3: knockdown of ALDOA with siRNA inhibited CRC cell proliferation and metastasis. (A, B) The qRT-PCR and western blotting were used to detect the knockdown efficiency of si-ALDOA. (C) CCK-8 assays assessed the reduction in CRC cell proliferation induced by si-ALDOA. (D) Wound healing assays examined the impaired migration ability of CRC cells caused by si-ALDOA. (E) Transwell assays evaluated the suppression of CRC cell invasive and migrative ability. ^∗^*P* < 0.05, ^∗∗^*P* < 0.01, and ^∗∗∗^*P* < 0.001. Figure S4: knockdown ALDOA inhibited CRC tumor growth in vivo. (A) Images of CRC xenograft tumors on male nude mice. (B-D) The size and weight of male mouse xenograft models were recorded. Volume = (length × width^2^)/2. (E) No significant difference in body weight was observed between the two groups. ^∗^*P* < 0.05, ^∗∗^*P* < 0.01, and ^∗∗∗^*P* < 0.001. Figure S5: ALDOA plasmid increased CRC cell phenotype on proliferation and metastasis. (A, B) The overexpression efficiency of pcDNA-ALDOA was verified by qRT-PCR and western blotting analyses. (C) The enhancement of CRC cell proliferation induced by pcDNA-ALDOA was detected by CCK-8 assay. (D, E) The effect of overexpressed ALDOA on the migration and invasion of CRC cells was examined by wound healing and transwell assays. ^∗^*P* < 0.05, ^∗∗^*P* < 0.01, and ^∗∗∗^*P* < 0.001.
