# Supplementary material for: Aldolase A Promotes Colorectal Cancer Progression through Targeting COPS6 and Regulating MAPK Signaling Pathway
Source: Dis Markers. 2023 Jul 6;2023:1702125. doi: 10.1155/2023/1702125 (PMC10344634; doi:10.1155/2023/1702125)
Supplement: Supplementary Materials — Figure S1: the expression of ALDOB and ALDOC in CRC tissues. (A) Western blotting and (B, C) qRT-PCR assays were used to assess the mRNA and protein levels of ALDOB and ALDOC in 24-paired CRC tissues. Figure S2: ALDOA had no significant effect on cell apoptosis. CRC cell apoptosis level was evaluated by (A, C) flow cytometry and (B, D) western blotting. Figure S3: knockdown of ALDOA with siRNA inhibited CRC cell proliferation and metastasis. (A, B) The qRT-PCR and western blotting were used to detect the knockdown efficiency of si-ALDOA. (C) CCK-8 assays assessed the reduction in CRC cell proliferation induced by si-ALDOA. (D) Wound healing assays examined the impaired migration ability of CRC cells caused by si-ALDOA. (E) Transwell assays evaluated the suppression of CRC cell invasive and migrative ability. ∗P < 0.05, ∗∗P < 0.01, and ∗∗∗P < 0.001. Figure S4: knockdown ALDOA inhibited CRC tumor growth in vivo. (A) Images of CRC xenograft tumors on male nude mice. (B-D) The size and weight of male mouse xenograft models were recorded. Volume = (length × width2)/2. (E) No significant difference in body weight was observed between the two groups. ∗P < 0.05, ∗∗P < 0.01, and ∗∗∗P < 0.001. Figure S5: ALDOA plasmid increased CRC cell phenotype on proliferation and metastasis. (A, B) The overexpression efficiency of pcDNA-ALDOA was verified by qRT-PCR and western blotting analyses. (C) The enhancement of CRC cell proliferation induced by pcDNA-ALDOA was detected by CCK-8 assay. (D, E) The effect of overexpressed ALDOA on the migration and invasion of CRC cells was examined by wound healing and transwell assays. ∗P < 0.05, ∗∗P < 0.01, and ∗∗∗P < 0.001. [file 1702125.f1.docx]

**Supplementary figures**


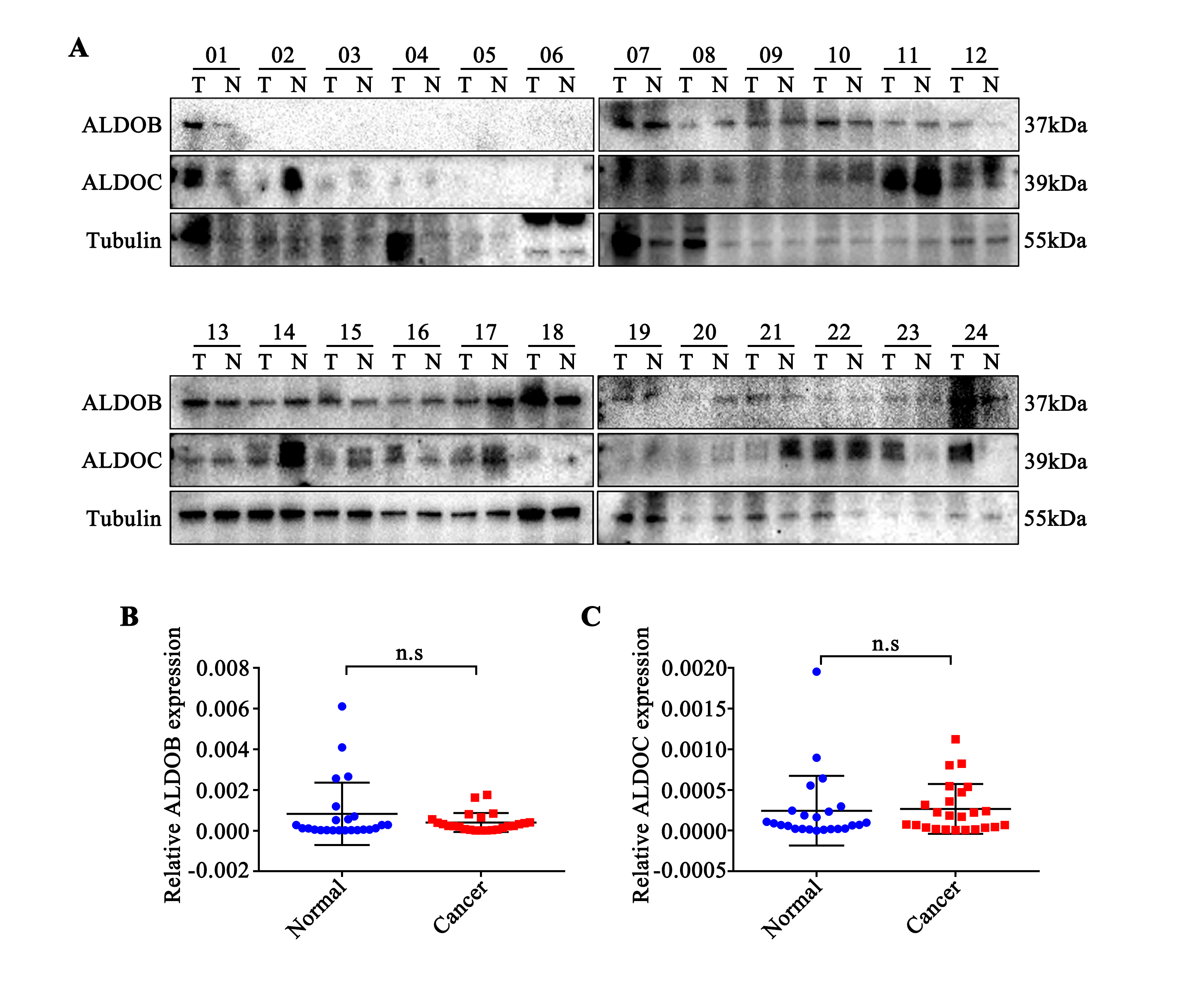


Figure S1: The expression of ALDOB and ALDOC in CRC tissues. (A) Western blotting and (B, C) qRT-PCR assays were used to assess the mRNA and protein levels of ALDOB and ALDOC in 24-paired CRC tissues.


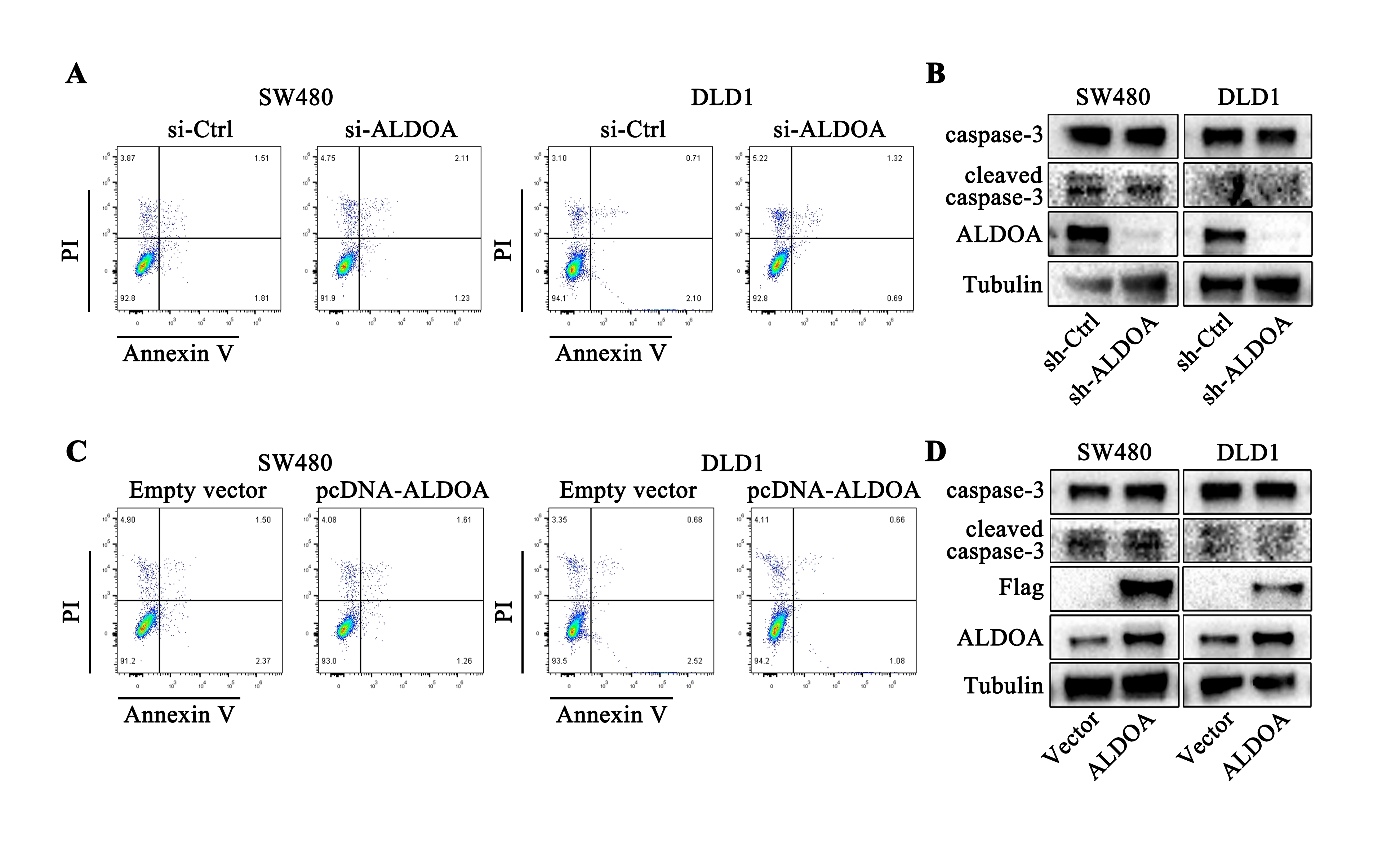


Figure S2: ALDOA had no significant effect on cell apoptosis. CRC cell apoptosis level was evaluated by (A, C) flow cytometry and (B, D) western blotting.


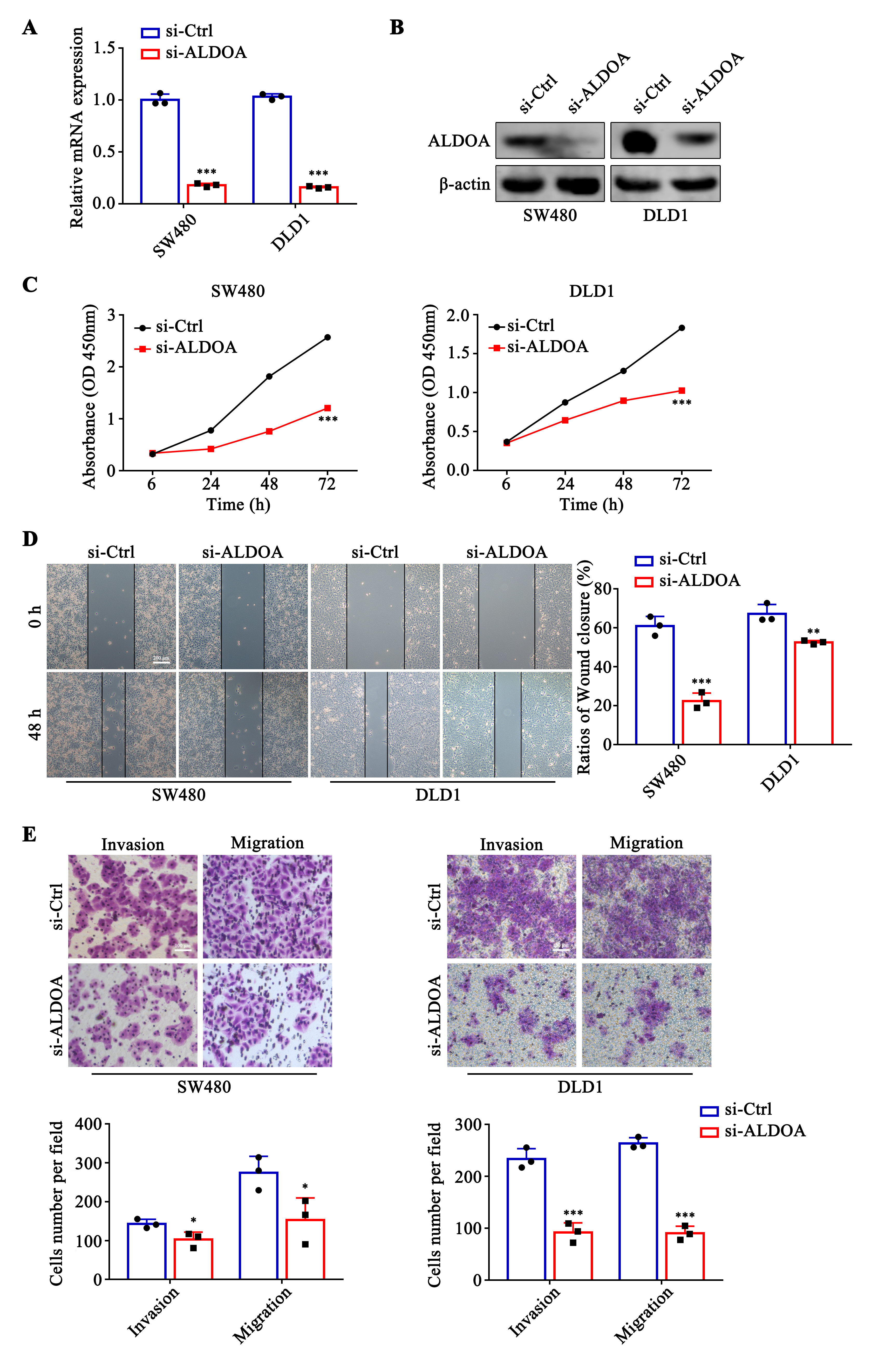


Figure S3: Knockdown of ALDOA with siRNA inhibited CRC cell proliferation and metastasis. (A, B) The qRT-PCR and western blotting were used to detect the knockdown efficiency of si-ALDOA. (C) CCK-8 assays assessed the reduction in CRC cell proliferation induced by si-ALDOA. (D) Wound healing assays examined the impaired migration ability of CRC cells caused by si-ALDOA. (E) Transwell assays evaluated the suppression of CRC cell invasive and migrative ability. *P＜0.05, **P＜0.01, ***P＜0.001.


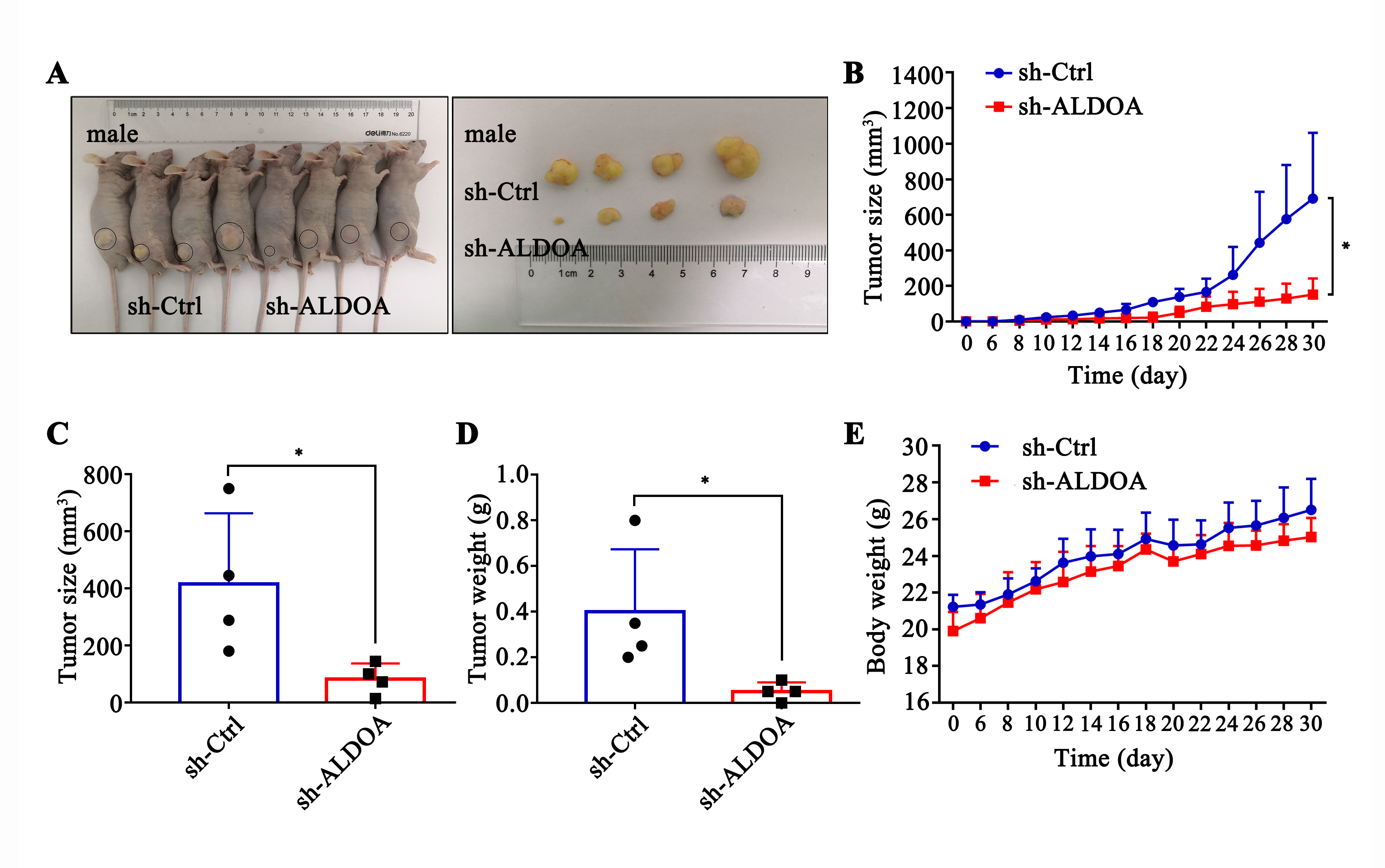


Figure S4: Knockdown ALDOA inhibited CRC tumor growth in vivo. (A) Images of CRC xenograft tumors on male nude mice. (B-D) The size and weight of male mouse xenograft models were recorded. Volume=length×width^2^/2. (E) No significant difference in body weight was observed between the two groups. *P＜0.05, **P＜0.01, ***P＜0.001.


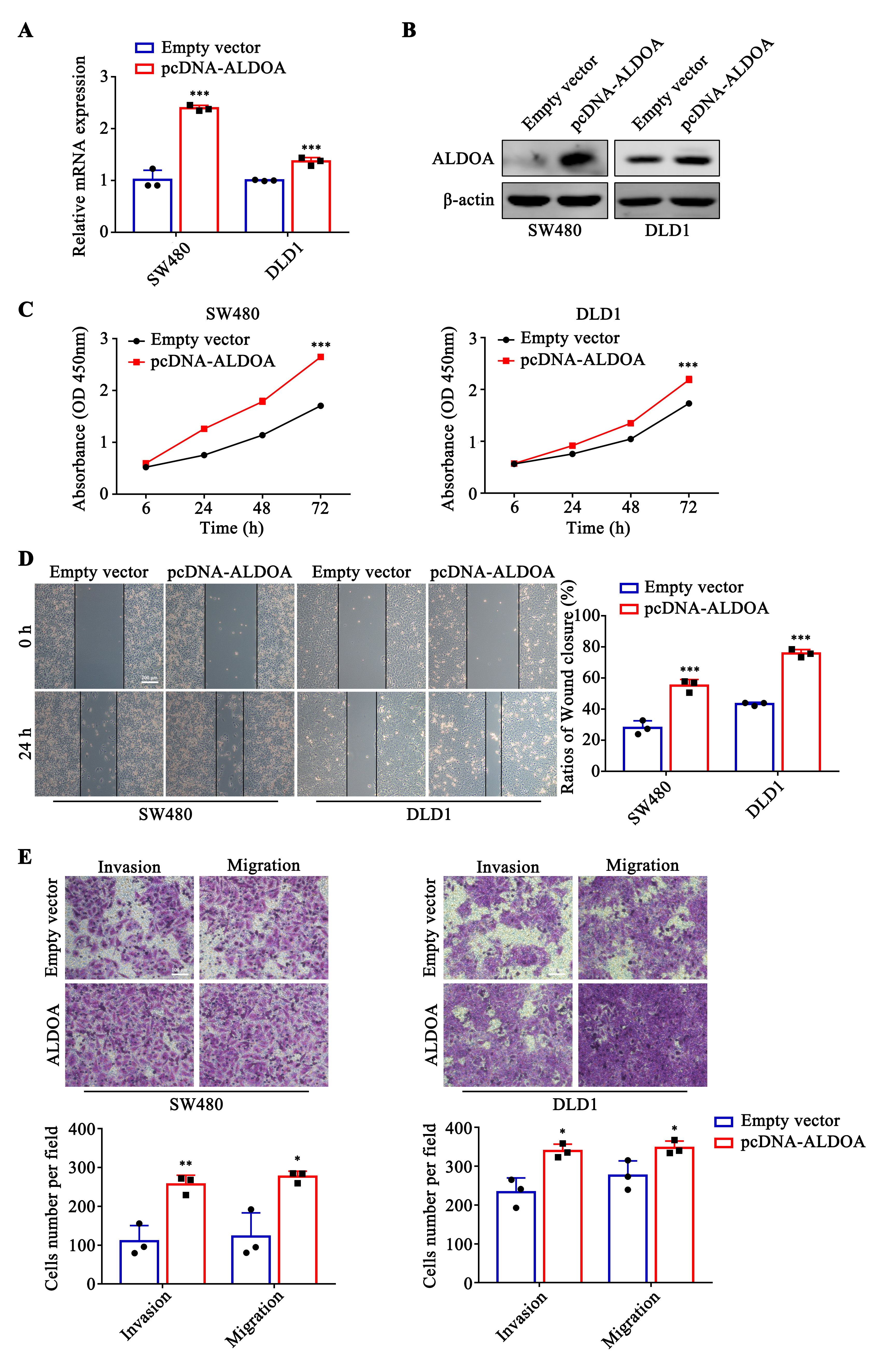


Figure S5: ALDOA plasmid increased CRC cell phenotype on proliferation and metastasis. (A, B) The overexpression efficiency of pcDNA-ALDOA was verified by qRT-PCR and western blotting analysis. (C) The enhancement of CRC cell proliferation induced by pcDNA-ALDOA was detected by CCK-8 assay. (D, E) The effect of overexpressed ALDOA on the migration and invasion of CRC cells was examined by wound healing and transwell assays. *P＜0.05, **P＜0.01, ***P＜0.001.
